# RT-ViT: Real-Time Monocular Depth Estimation Using Lightweight Vision Transformers

**DOI:** 10.3390/s22103849

**Published:** 2022-05-19

**Authors:** Hatem Ibrahem, Ahmed Salem, Hyun-Soo Kang

**Affiliations:** 1 Department of Information and Communication Engineering, School of Electrical and Computer Engineering, Chungbuk National University, Cheongju-si 28644, Korea; hatem@cbnu.ac.kr (H.I.); ahmeddiefy@cbnu.ac.kr (A.S.); 2 Electrical Engineering Department, Faculty of Engineering, Assiut University, Assiut 71515, Egypt

**Keywords:** monocular depth estimation, convolutional neural networks, vision transformers, real-time processing

## Abstract

The latest research in computer vision highlighted the effectiveness of the vision transformers (ViT) in performing several computer vision tasks; they can efficiently understand and process the image globally unlike the convolution which processes the image locally. ViTs outperform the convolutional neural networks in terms of accuracy in many computer vision tasks but the speed of ViTs is still an issue, due to the excessive use of the transformer layers that include many fully connected layers. Therefore, we propose a real-time ViT-based monocular depth estimation (depth estimation from single RGB image) method with encoder-decoder architectures for indoor and outdoor scenes. This main architecture of the proposed method consists of a vision transformer encoder and a convolutional neural network decoder. We started by training the base vision transformer (ViT-b16) with 12 transformer layers then we reduced the transformer layers to six layers, namely ViT-s16 (the Small ViT) and four layers, namely ViT-t16 (the Tiny ViT) to obtain real-time processing. We also try four different configurations of the CNN decoder network. The proposed architectures can learn the task of depth estimation efficiently and can produce more accurate depth predictions than the fully convolutional-based methods taking advantage of the multi-head self-attention module. We train the proposed encoder-decoder architecture end-to-end on the challenging NYU-depthV2 and CITYSCAPES benchmarks then we evaluate the trained models on the validation and test sets of the same benchmarks showing that it outperforms many state-of-the-art methods on depth estimation while performing the task in real-time (∼20 fps). We also present a fast 3D reconstruction (∼17 fps) experiment based on the depth estimated from our method which is considered a real-world application of our method.

## 1. Introduction

Depth estimation is a critical task in a variety of computer vision applications, including 3D scene reconstruction from 2D images, medical 3D imaging, augmented reality, self-driving cars and robots, and 3D computer graphics and animations. The recent advances in depth estimation research have shown the effectiveness of the convolutional neural networks (CNNs) in performing such a task [1,2,3,4,5,6,7,8,9,10,11,12,13,14,15]. The encoder-decoder CNN architectures are the most used architectures in the dense prediction tasks [2,3,4,5,6,7,8,9,10,11,12] (image-like predictions such as semantic segmentation and depth estimation). The encoder stage compresses the input image to a latent small representation that consists of rich feature maps and the decoder stage uncompress the feature by a sequence of deconvolutional or up-sampling layers in order to obtain pixel-wise predictions. Although the convolutional-based architectures could perform the depth estimation task efficiently, the convolution algorithm itself lacks the global awareness of the image content as it depends on a limited window (filter) with a small size (3×3 or 5×5 are the most common filter sizes). Learning of the global content of an image or a sequence of images is very useful for more accurate predictions in all computer vision tasks, so many attention algorithms such as RNN [16] or LSTM [17] are employed in order to provide a relationship between the images in a sequence of images but those methods are usually computationally expensive and have a limited attention length. Vaswani et al. [18] proposed a solution for those problems in the natural language processing tasks by composing the multi-head self-attention (MSA) mechanism which aims to get the relation between each word and all of the other words in a word sequence. The MSA mechanism calculates a weighted average of the extracted features of a word with the weight value is proportional to the similarity between the extracted features from a pair of words. The input sequence of tokens is projected using three learnable matrices $W_{Q}$, $W_{k}$, and $W_{V}$ to extract features of the query (Q), the key (K), and the value (V) representations using a normalized softmax function. The attention in MSA is performed independently which enables the parallelization of the process.

The vision transformers (ViT) research was proposed by Dosovitskiy et al. [19] in which they employed the MSA mechanism in the image classification task. They dealt with the image as a sequence of patches that have inter-relationships which the model aims to learn. First, they split the image into patches which are embedded into 1D vector each and the position of each patch is also encoded into the embedded patch then they fed the embedded patches to a transformer encoder which consists of a sequence of identical MSA mechanisms to learn the cross-relation between patches for better understanding of the global content of the image. The ViT outperformed the state-of-the-art CNN architectures in the image classification task on ImageNet benchmark [20] and JFT-300M Google’s private dataset [21]. We employ ViT for the depth estimation task showing that it outperforms the conventional architectures on depth estimation. We state our contribution in this paper as follows:

- We propose efficient architectures for accurate depth estimation based on a ViT encoder and convolutional decoder architectures;
- We show that the proposed model can attain a high speed of frame processing (∼20 fps) which is convenient for real-time depth estimation;
- We propose architectures with different combinations composed of four ViT-based encoder architectures and four different CNN-based decoder architectures;
- We show that the ViT encoder can extract much more representative features and so a better depth estimation accuracy than the fully convolutional-based architectures and even some ViT-based architectures by comparison.

**Figure 1:**
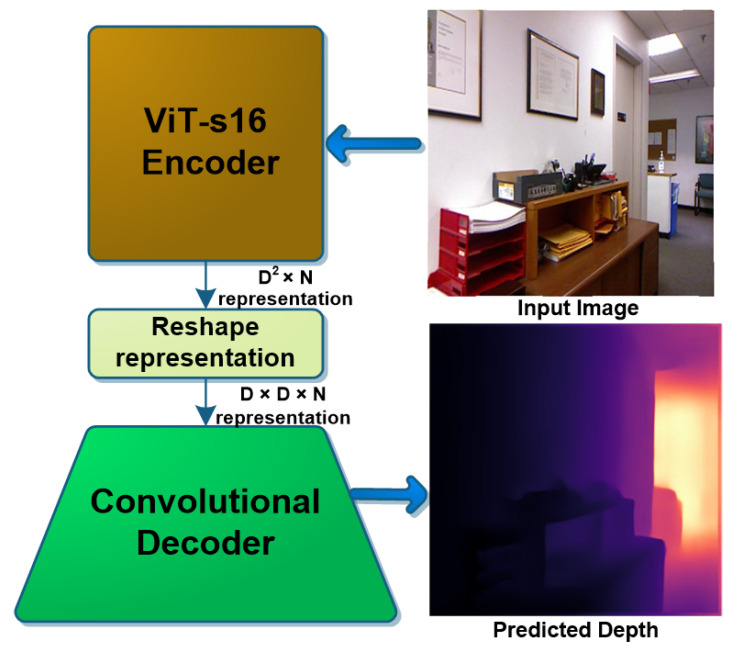
Overview of the proposed encoder-decoder architecture using a ViT encoder and a CNN decoder.

An overview of the proposed method is shown in Figure 1. The order of this paper is as follows. Section 2 presents the related work and Section 3 shows the details of the proposed method and architectures. Section 4 and Section 5 discuss the benchmarks for training and test, and present the experiments performed in this research, respectively. Section 6, Section 7 and Section 8 discuss the evaluation metrics, the results obtained, and comparisons with the recent state-of-the-art methods, respectively. Section 9 presents an application of the proposed method in 3D reconstruction. Section 10 shows the paper conclusions and Section 11 discusses the future work based on this research.

## 2. Related Work

Several CNN architectures were introduced to solve the problem of depth estimation. Eigen et al. [1] was one of the first deep learning approaches to perform depth estimation. They [1] proposed a two stages CNN architecture, the first stage predicts a coarse depth map and the second stage is a refinement stage for the output from the first stage in order to obtain an accurate depth map. This approach [1] proved the possibility of monocular depth estimation using CNN. Li et al. [2] proposed an encoder-decoder CNN architecture employing the conditional random fields (CRFs) to directly learn the mapping from RGB image to the depth map. Liu et al. [3] also proposed a similar encoder-decoder architecture with CRF for super-pixel segmentation in order to refine the depth map. Gan et al. [4] proposed an encoder-decoder architecture to learn the global context of the image for better depth estimation using an affinity layer and vertical max pooling. Xu et al. [5] proposed a multi-scale CNN architecture with a cascade of CRFs to fuse the best values of the depth. Kuznietsov et al. [6] proposed a CNN architecture with an image alignment loss to improve the depth predictions. Fu et al. [7] employed space increasing discretization to perform a pixel-wise classification to obtain the depth map. Cao et al. [8] also considered the depth estimation problem as a classification problem by converting the continuous depth values into discrete values then predicting the depth map as a probability distribution. Cao et al. [9] also proposed a depth estimation technique based on a pre-training stage on the relative depth from stereo-depth then fine-tuning the CNN network on the monocular depth. Niklaus et al. [10] proposed a semantic-aware CNN for depth estimation based on semantic segmentation which enhanced the depth estimation of object boundaries. Zuo et al. [11] proposed depth enhancement techniques using a multi-scale intensity guidance CNN with residual connection to improve the depth prediction. Wu et al. [12] proposed PhaseCam3D in which they employed an encoder-decoder architecture to learn the optimal phase mask for the depth estimation. Lee et al. [13] proposed a CNN-based method namely From big to small (BTS) which utilizes local planar guidance layers at different scales in the decoder stage that guides the feature maps to accurate depth predictions. We also provided challenging depth estimation results in previous research [14,15] in which we eliminate the complexity of the decoder in the encoder-decoder CNN architecture using depth-to-space (pixel-shuffle) image reconstruction. Although the previously stated methods attained relatively good results, the estimated depth in most of the stated methods has blurry results especially at the borders of the objects in the scene due to the inefficient encoding and decoding stages due to the local learning scheme naturally provided by the convolution algorithm. We solve this problem by using ViT encoder which has global attention for all pixels in the scene instead of the local attention in the case of the convolutional-based methods. Recently, there are depth estimation methods that adopt a vision transformer architecture such as Adabins [22] which applied a mini-ViT on the output features obtained from a CNN encoder-decoder as a refinement stage applying what the author called depth bins. Yang et al. [23] proposed TransDepth which is a transformer-based depth estimation employing ResNet [24] to extract the input image features then applying transformer layers to obtain much deeper and more generalized features which are added to the upsampled features from different layers of the ResNet [24] to obtain better depth estimation accuracy. Ranftl et al. [25] adopted a ViT encoder and a different scale reassembling decoder to obtain image-like representations for depth estimation. Although the previously mentioned transformer-based methods are efficient, they lack the real-time processing feature that we propose in this paper using small and tiny ViT architectures for the encoder and we empirically select not only the best architecture in accuracy but the fastest CNN decoder among three different architectures, we compare our proposed depth estimation method with all the previously mentioned methods as we will show in the results section. Our proposed method differs from DPT [25] which is a recent vision transformer-based depth estimation method. DPT [25] adopts a different encoder since the authors used the large ViT (ViT-L) with 24 transformer layers however in our implementation we use far simpler transformers with 12, 6, and 4 transformer layers as we proved that the excess use of transformer layers is redundant in some cases where the ViT-s16 and ViT-t16 attained better error values and accuracies. The decoder architecture of DPT is also quite a different approach since they use a three-stage reassemble operation using convolutional layers in which they resample the transformer output, concatenate the outputs from different transformer layers, they project them all to get the dense prediction; however, in our approach we experiment four different decoders with three different up-sampling strategies to explore the best decoder, which is partially dependent on the dataset.

## 3. Proposed Method

**Figure 2:**
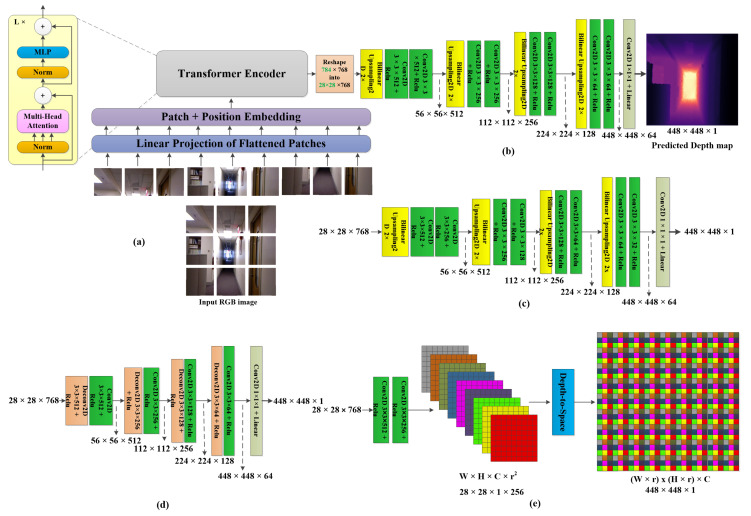
The proposed architectures: (**a**) The ViT encoder. (**b**) The CNN decoder with a progressive bilinear up-sampling architecture (US1), (**c**) The second CNN decoder with a progressive bilinear up-sampling architecture with a lower number of kernels in the second convolution of each block (US2), (**d**) The CNN decoder with the deconvolution up-sampling architecture (Deconv), and (**e**) The CNN decoder with the depth-to-space up-sampling architecture (DS).

The proposed method, shown in Figure 2, depends mainly on a ViT encoder architecture and a CNN decoder. We try different numbers of transformer layers at the encoder as well as different configurations at the decoder as shown in the following subsections.

### 3.1. ViT Encoder Architectures

We train different versions of ViT architectures that differ in the number of transformer layers. The main architecture of ViT-b16 consists of, firstly, the linear projection of the 16 × 16 patches of the input image from 2D patches into 1D vectors each. Secondly, patch and position embedding of 1D projected vectors of the input image patches. The embedding is performed by multiplying each 1D vector of the projected patches by its position in the image. Finally, the embedded vectors are fed to the transformer encoder which is a series of twelve normalization+ multi-head attention+ multi-layer perceptron (MLP) blocks with skip connections (namely transformer encoder) as shown in Figure 2a in the content of the transformer encoder. The transformer aims to parallelly attend to the objects in the image based on the MSA mechanism and the learnable weights of MLP. MSA uses scaled dot-product attention which can be stated as in Equation (1):

(1) $$Attention\left(Q,K,V\right)=softmax\left(\frac{QK^{T}}{{\sqrt{d}}_{k}}\right)V,$$

where *Q*, *K*, and *V* are query, Key, and value dimensional vectors respectively. $d_{k}$ is the variance of the product $QK^{T}$, which has a zero mean. So in order to normalize the product, it is divided by the standard deviation ${\sqrt{d}}_{k}$ where $d_{k}$ is the variance of the product. The softmax function is used to translate the scaled dot product into a score of attention. The MSA mechanism is the key module of the transformers which enables parallel attention to the global image content. We use an input image size of 448×448 for NYU depthv2 so the size of the features inside all the transformer encoder layers is 785×768, this first feature from the 1st dimension is excluded to obtain 784×768 to be reshaped to a 2D features of size 28×28×768. The reshaped features are fed to the designed decoders which have three different up-sampling techniques of the features in order to construct the depth map. We also train two reduced versions of ViT-b16 which we call small vision transformer (ViT-s16) and tiny vision transformer (ViT-t16) in order to obtain cheaper computational cost and so lesser processing time.

### 3.2. CNN Decoder Architectures

We try four different configurations of the decoder architecture. The first designed decoder consists of three convolutional layers with RELU activations followed by a bilinear up-sampling2D layer followed by two convolutional layers with the depth of the features is reduced through the network (512→256→128→64) as shown in the decoder in Figure 2b. A 1×1×1 convolution with linear activation is added at the end of the decoder stage to project the final 64 feature maps of size 448×448 into a 1 feature of the same size which represents the depth map prediction with the same size of the input image, we call that encoder architecture “US1” because it uses mainly the bilinear upsampling for. Slightly different convolutional filter numbers are employed to lower the number of computations in the decoder stage, we replaced the second convolutional layer in each block of the first architecture with half of the number of the filters of the first convolution in the block as shown in Figure 2c, we call that decoder architecture “US2”. The third decoder architecture employed consists of a sequence of deconvolution (transpose convolution) layers plus convolutional layers both with Relu activations. The deconvolution layers are configured as 2×2 kernels with a stride of 2 to double the size of the input and the convolutional layers are configured with 3×3 kernels with a stride of 1. The feature depth through the network is 512→256→128→64 with a 1×1 convolution as the last layer to predict the depth map as shown in Figure 2d, we call that decoder architecture “Deconv”. The fourth decoder architecture is a simple depth-to-space (DS) module inspired from the image super-resolution method in [26] which rearrange the pixels in the reshaped small feature maps with a size of $W\times H\times C\times r_{2}$ in order to construct a larger map of a size rW×rH×C through a learnable aggregation process as shown in the following equation:

(2) $$M_{Depth}=Rearran\left(W_{L}\times f^{L-1}\left(M_{features}\right)+b_{L}\right),$$

where $M_{Depth}$ and $M_{features}$ are the constructed depth map and the reshaped features respectively, $W_{L}$ and $b_{L}$ are the learnable weights and biases of the process. Rearran is a rearrangement function. First, we reduce the number of filters of the reshaped representation from 28×28×728 to 28×28×256 using two 3×3 convolutional layers with filter count of 512 and 256 sequentially. The 28×28×256 representation is fed to the depth-to-space module to obtain the final depth map with a size of 448×448×1 as shown in Figure 2e, we call that decoder architecture “DS”.

The depth map is learned through depth regression using an image construction loss (*CL*), we employ Huber loss as a construction loss with δ=1 as shown in Equation (3):

(3) $$CL=\left\{\begin{array}{c} \frac{1}{N}\sum_{i=1}^{N}{\left(Y_{i}-{\hat{Y}}_{i}\right)}^{2},\;\mathrm{if}\left|Y-\hat{Y}\right|<\delta \\ \frac{\delta }{2N}\sum_{i=1}^{N}\left(\right|Y_{i}-{\hat{Y}}_{i}\left|-\frac{1}{2}\delta \right),\;\mathrm{otherwise} \end{array}\right.$$

where *N* and *i* are the pixels count and the pixel iterator, respectively. *Y* is the ground truth pixel value and $\hat{Y}$ is the predicted depth value, while the threshold value, δ, is selected as 1 since empirically it speeds up the training process. Huber loss performed better than L 1 (Mean Absolute Error) and L2 (Mean Squared Error) separately by experiments. The proposed encoder-decoder architectures are trained end-to-end using the loss function (3) and the weights of the ViT layers in the encoder and the convolutional layers in the decoder are updated simultaneously by the back-propagation process.

## 4. Benchmarks for Training and Validation

We separately train and test the proposed method on two benchmarks; NYU-depthV2 [27] and CITYSCAPES [28]. NYU-depthV2 [27] dataset consists of images and their corresponding depth maps from indoor scenes such as bedrooms, living rooms, bathrooms, kitchens, etc. The dataset consists of 1449 densely labeled images and 407,024 pseudo labeled and unlabeled images. We train and test our method on the densely labeled images since the pseudo labeled images have much noise and invalid depth values that will negatively affect the model’s accuracy. We adopt the common dataset split which split the dataset into 795 training images and 654 validation images, we resize the original image and depth map size of the dataset which is 640×480 into 448×448 to speed up the training process. We also evaluate the model with the same image size. CITYSCAPES [28] is another dataset used for depth estimation; it consists of driving scene images captured in 50 different urban cities in Germany. It consists of 2975 pairs of training images (RGB images and their corresponding disparity maps) and 500 pair of images for validation. We directly predict the disparity map as it represents the depth since the relationship between the disparity and the depth is linear (depth=Baseline×focal_length/disparity ) and both the baseline and the focal length are known and almost are constants for all images. We resize the original image size of the RGB images and disparity maps of CITYSCAPES is 2048×1024 to 640×320 which corresponds to one-third of the original image size to speed up the training and the inference while maintaining an acceptable image size. The features extracted from the encoder is 800×768 is reshaped to 20×40×768. We also evaluate the model on the 500 validation images of the dataset.

## 5. Experiments

We train the proposed method using different combinations between the proposed encoder and decoder architectures. The first trial was training a ViT-b16 encoder with the CNN decoder architecture in Figure 2b, then we tried a smaller ViT architecture, ViT-s16 with US2 decoder in Figure 2c and the smallest encoder architecture ViT-t16 with the three architectures (US2, Deconv., DS) in Figure 2c–e respectively, each one was trained separately. ViT-t16 was empirically the best encoder architecture for the real-time processing thanks to the reduced ViT architecture however the decoder architecture in use has a non-neglected impact on both the accuracy and the speed of the depth estimation task. The results obtained from all of those experiments are reported in the next section (Results). Regarding the training details, we initialized the original ViT-b16 transformer model with ImageNet-1K+ImageNet-21k weights from the original ViT research [19] which allows fast fitting of the model, for the small ViT model (ViT-s16) and the tiny ViT model (ViT-t16), we also initialize the original ViT-b16 with ImageNet-1K+ImageNet-21k weights then we construct the model using the embedding layers and the first pre-initialized four transformer layers. We add the layer-normalization after the transformer layers, finally, we reshape the representation and feed it to the designed decoders. All models are trained for 1500 epochs on two desktop computers with Nvidia TITAN RTX GPU and Nvidia RTX3090 GPU using the Tensorflow Keras environment. Adam optimizer is used for models optimization during training. The models are tested under the same environment on the Nvidia RTX3090 GPU only.

## 6. Evaluation Metrics

To evaluate the depth estimation results, we used the common standard metrics for depth estimation which are:

- The delta accuracy $\delta_{i}$ of $y_{i}$: $max(\frac{y}{\hat{y}},\frac{\hat{y}}{y})=\delta <t$ for threshold values $t=1.25,1.25^{2},1.25^{3}$;
- The absolute relative error (Abs_Rel): $\frac{1}{N}\sum_{i}^{N}\frac{|y_{i}-\hat{y_{i}}|}{y}$;
- The squared relative error (Sq_Rel): $\frac{1}{N}\sum_{i}^{N}\frac{\left|\right|y_{i}-\hat{y_{i}}{\left|\right|}^{2}}{y}$;
- The root mean squared error ($RMSE_{log}$): $\sqrt{\frac{1}{N}\sum_{i}^{N}{\left(log\left(y\right)-log\left(\hat{y}\right)\right)}^{2}}$,

where *y* and $\hat{y}$ are the ground truth and predicted pixel values and n is the total number of the pixels in the depth map.

## 7. Results

**Figure 3:**
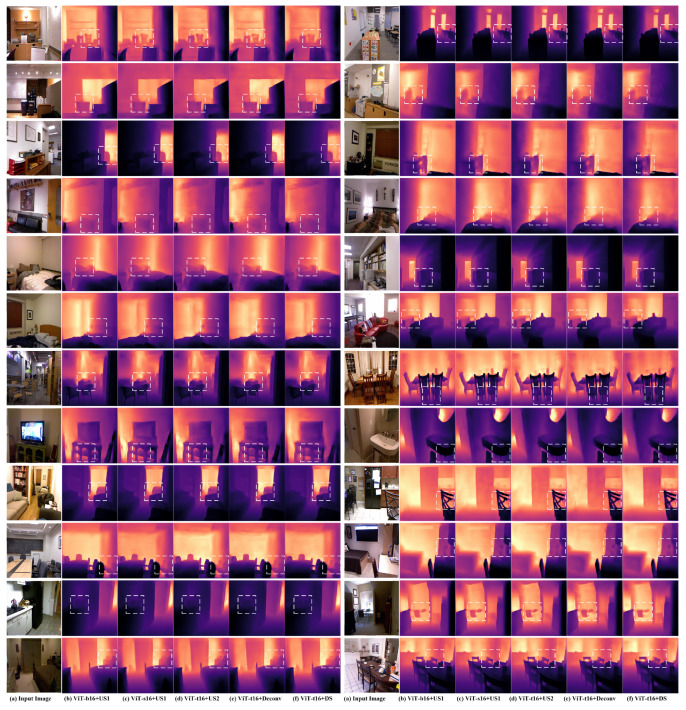
Sample results obtained from the proposed architectures on Cityscape validation-set: (**a**) the input image, (**b**–**f**) are the depth estimation results obtained from the architecture mentioned below the column, (**b**) ViT-b16 encoder and US1 decoder, (**c**) ViT-s16 encoder and US1 decoder, (**d**) ViT-t16 encoder and US2 decoder, (**e**) ViT-t16 encoder and the Deconv decoder, and (**f**) ViT-t16 encoder and (depth-to-space) DS decoder. The regions inside the dashed white boxes include slightly obvious differences between the results of the proposed architectures on the same scene.

We evaluate the proposed architectures on the NYU-depthv2 test set, the CITYSCAPES validation set, and the CITYSCAPES test set. We reported the results for different combinations of the encoder architectures of ViT and the CNN decoder architectures. Table 1 shows the evaluation results on each one of the datasets. on NYU-depthV2, ViT-b16+US1 architecture obtained the lowest error values and the best delta accuracies but its speed is relatively slow (10.97 fps). For Vit-s16, it attains worse errors and delta accuracies but it attains more speed in processing (15.87 fps). The architectures with tiny ViT encoder model ViT-t16 attained the fastest processing speed but with slightly higher errors and lower accuracies, whereas ViT-t16+DS is the fastest model with a speed of 20.83 fps, which is convenient with real-time applications. The other tiny ViT models (ViT-t16+Dceonv. and ViT-t16+US2) attain better accuracy and error values than those for the ViT-16+DS and also have an acceptable speed of processing (18.18 fps and 19.61 fps respectively). Figure 3 shows the high quality of sample results obtained from the proposed architecture on the NYU-depthv2 dataset. The quality of the results can almost be considered similar with very small differences (the error and delta accuracy values are slightly different as shown in Table 1) that can be seen when zooming in the dashed white boxes (regions with slightly obvious depth differences).

More encoder-decoder combinations were tested on the CITYSCAPES validation-set and test-set as shown in Table 1. The results showed that ViT-t16+Deconv has the best performance even better than ViT-b16+US1, which has many more transformer layers. We think that this comes from the nature of the data since the disparity map in the case of CITYSCAPES is sparser than the depth map in the case of NYU-depthv2 (which is dense). The sparse representation makes the accuracy better since the network during evaluation is only tested on the valid pixels only and the invalid pixels (which are many) are excluded. The results on Cityscapes prove that the ViT-b16 and ViT-s16 have many redundant layers since four transformer layers (ViT-t16) are just enough to learn the encoding process fast and accurately. The deconvolution-based decoder is also the most efficient one in constructing the depth map from the encoded representation in that case.

**Figure 4:**
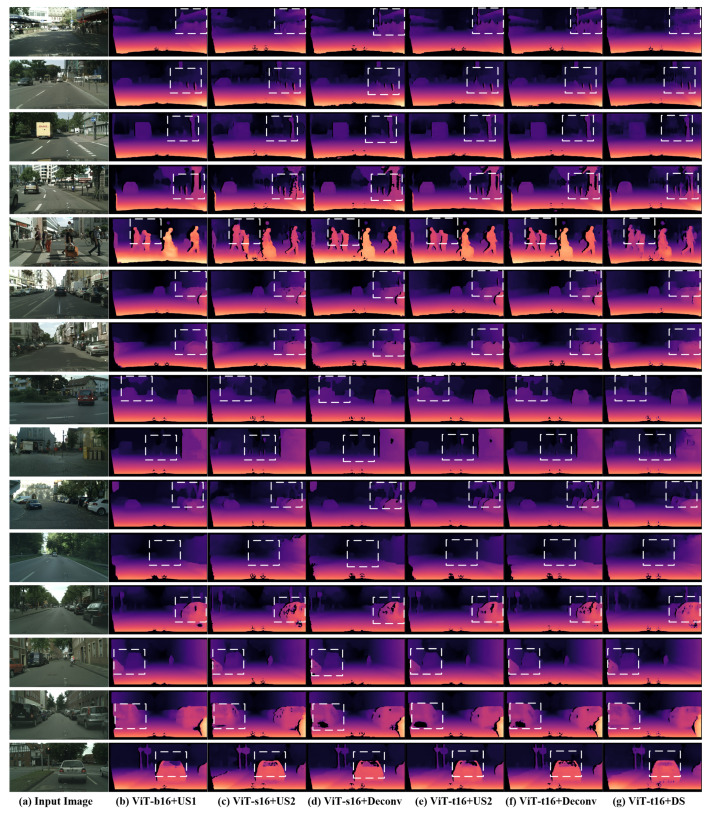
Sample results obtained from the proposed architectures on Cityscape validation-set: (**a**) the input image, (**b**–**g**) are the depth estimation results obtained from the architecture mentioned below the column, (**b**) ViT-b16 encoder and US1 decoder, (**c**) ViT-s16 encoder and US2 decoder, (**d**) ViT-s16 encoder and the Deconv decoder, (**e**) ViT-t16 encoder and US2 decoder, (**f**) ViT-t16 encoder and the Deconv decoder, and (**g**) ViT-t16 encoder and (depth-to-space) DS decoder. The regions inside the dashed white boxes include slightly obvious differences between the results of the proposed architectures on the same scene.

We also evaluated the proposed architectures on the Cityscapes test-set. The test-set contains 1525 pairs of RGB/disparity images. ViTt-16+DS showed the best performance in terms of speed (12.5 fps) and accuracy ($\delta_{3}$ = 0.9646) however, the other combinations showed average to good accuracy and speed as predicted. Table 1 shows the evaluation results on the CItyscapes test-set, reporting the depth errors and the delta accuracies on the five architectures (ViT-b16+US1, ViT-s16+US2, ViT-s16+Deconv, ViT-t16+US2, ViT-t16+Deconv, and ViT-t16+DS). Figure 4 shows qualitative results on the CITYSCAPES validation-set obtained by the previously mentioned five architectures with some small differences in the predictions of the same scene bounded by the dashed white boxes. Appendix A shows extra results obtained by our architectures on CITYSCAPES test-set.

## 8. Comparison with the SOTA Methods

We compared the proposed architectures with the state-of-the-art (SOTA) methods in depth estimation. Table 2 compares two of the proposed architectures (ViT-b16+US1 and ViT-t16+DS) with CNN-based and ViT-based methods. The two architectures (ViT-b16+US1 and ViT-t16+DS) outperform the SOTA methods in terms of absolute relative error (Abs_Rel), $RMSE_{log}$, and the three delta accuracies ($\delta_{1}$, $\delta_{2}$, and $\delta_{3}$ ), which shows the excellent performance of the proposed method over all the SOTA methods. The proposed architectures not only outperform the recent CNN-based SOTA methods such as BTS [13], SDC-depth [29], and PhaseCam3D [12] but they also outperform DPT-Hybrid [25] and AdaBins [22] that are recent transformer-based methods that prove that our method exploits the vision transformer in a better way than those methods do.

**Figure 5:**
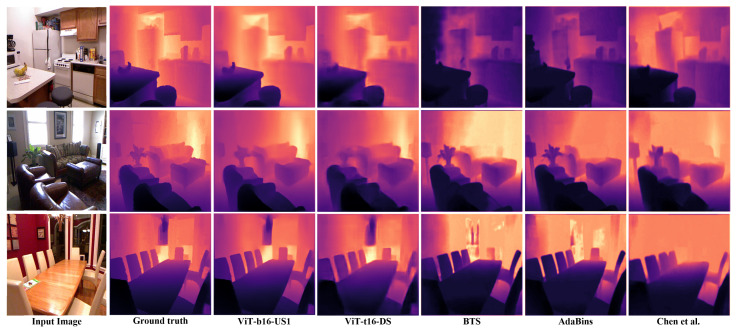
Quality comparison between the depth estimation results obtained by the proposed method (ViT-b16+US1 and ViT-t16+DS), BTS [13], AdaBins [22], and Chen et al. [1] on sample images from the NYU-depthv2 validation-set.

Table 3 compares also two architectures (ViT-t16+Deconv which is the best model in terms of accuracy and ViT-t16+DS which is the best in terms of speed) with conventional CNN-based methods on depth estimation. ViT-t16+Deconv is better than all the other SOTA methods in terms of Rel_Abs, $RMSE_{log}$, and the delta accuracies. The ViT-t16+DS architecture has a lower Abs_Rel and delta accuracies than those of Laina et al. [29,30] but it outperforms it in terms of $RMSE_{log}$ since $RMSE_{log}$ metric is less sensitive to the outliers and can scale down the effect of the outliers, this means that our model can predict more accurate depth maps than the other SOTA methods neglecting the effect of the outliers. ViT-t16+DS also outperforms the three other SOTA methods in terms of all the depth estimation metrics in the comparison. Figure 5 shows a quality comparison between our method (ViT-b16+US1 and ViT-t16+DS) and three other methods (BTS [13], AdaBins [22], and Chen et al. [1]) on samples from NYU-depthv2, it shows the good quality of the results obtained by our method that are so close to the ground truth depth maps, however, the results from the other method have some mispredicted parts in their predicted depth maps. The good achievement in this research is that we produce such good quality at high processing speed.

## 9. Fast 3D Reconstruction as an Application of the Proposed Method

We performed an experiment for fast 3D construction using the proposed method on the NYU-depthV2 dataset. We construct the point cloud from the estimated depth with our method and color the points using the colors in the RGB image. We directly estimate the 3D scene by estimating each point location from the depth and the pixel-RGB values following the equations of the point cloud estimation from RGB-D as follows:

(4) $$z=\frac{d}{Scale_{d}}$$

(5) $$x=\frac{(u-c_{x})*z}{f_{x}}$$

(6) $$y=\frac{(v-c_{y})*z}{f_{y}}$$

where *x*, *y*, and *z* represent the point location in the 3D space. *d* is the depth value, $Scale_{d}$ is the scale of the depth, *u* and *v* represent the pixel location in *x*-*y* coordinate. $c_{x}$ and $c_{y}$ represent the optical center in a pinhole camera model, $f_{x}$ and $f_{y}$ are the focal lengths of the camera’s lens in x and y axes. $Scale_{d}$, $c_{x}$, $c_{y}$, $f_{x}$, and $f_{y}$ are all fixed values given with NYU-depthV2 dataset. We employed ViT-t16+Deconv as it has a high delta accuracy ($\delta_{3}$ = 0.999) and a fast speed (19.6 fps) to perform fast construction of the point cloud. We used the same desktop computer as this for training and testing of our method for the 3D reconstruction task.

**Figure 6:**
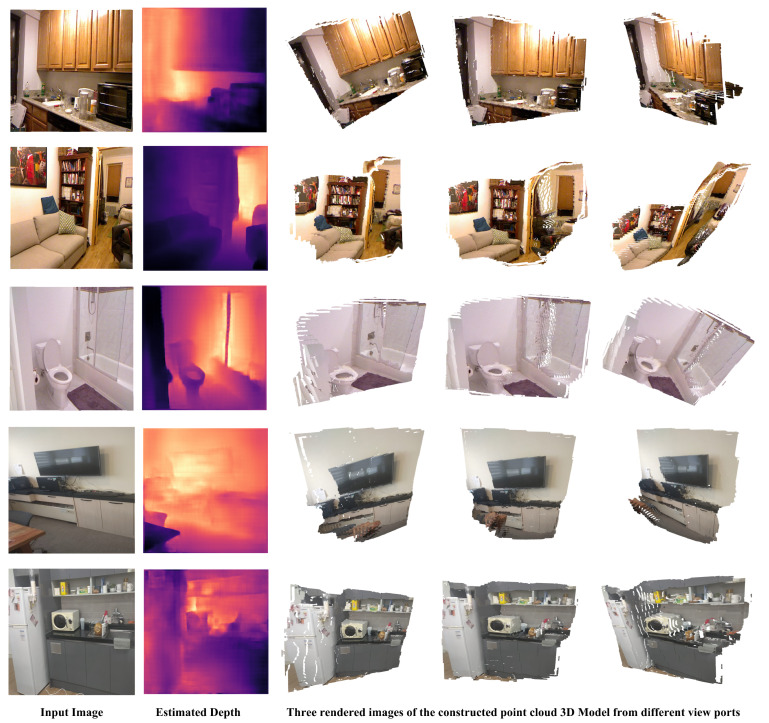
Three dimensional (3D) reconstruction results that were obtained using our method (ViT-t16+Deconv). The input image and the estimated depth for each sample are used to construct a point cloud following Equations (4)–(6). Three rendered images of each sample from different viewpoints are shown to the right of the estimated depth. The first three images (in the most left column) are samples from NYU depthV2 and the bottom two images are images taken from our laboratory.

Figure 6 shows sample 3D reconstruction results of images from NYU depthV2 and images taken from our laboratory. The reconstruction process takes 6 milliseconds and the prediction of the depth using our model (ViT-t16+Deconv) takes 52 milliseconds, after all the whole process including prediction of the depth from RGB image and the 3D reconstruction takes 58 milliseconds, which is equivalent to 17.2 fps, which is good enough for real-time 3D reconstruction from a single image considering the general complexity of the 3D construction process.

## 10. Conclusions

We proposed a real-time monocular depth estimation method that can efficiently learn the task of depth estimation thanks to the global image learning using attention provided by the vision transformers in the encoder stage. The obtained performances are not only good but also have the advantage of the fast processing speed (∼20 fps), which is convenient with real-time applications. The main contribution can be considered as the high processing speed of the tiny (ViT-t16) and small (ViT-s16) versions of the vision transformer, which is a great achievement since most of the methods which employ vision transformers are usually slow due to the extensive use of the transformer layers that require a large number of expensive computations (because of the use of the fully connected layers). We performed a reduction to the base transformer architecture which, in our case, has many redundant transformer layers that can be removed without having too much negative effect on the prediction’s accuracy. We also conducted an experiment on the fast 3D reconstruction based on the depth obtained from the proposed method which shows the effectiveness of our method in the 3D reconstruction task from a single RGB image in real-time. The proposed method in general is simple, however it can learn complex tasks as we showed for the depth estimation and the method can easily be extended for other computer vision tasks.

## 11. Future Work

The success of those reduced models of the vision transformers (i.e., ViT-s16 and ViT-t16) opens the door for more real-time transformer-based methods. The straightforward approach that can be built upon this work is real-time semantic segmentation using the same proposed architectures or slightly modified ones to fit the task. Another approach can exploit such lightweight models in more advanced tasks such as real-time object detection, which needs further work in developing the best technique to exploit those lightweight architectures in learning the object recognition and the bounding-box regression.

## Figures and Tables

**Table 1:** Evaluation of the proposed architectures with different combinations of the encoder and decoder configuration on the NYU-depthV2 test-set and the CITYSCAPES validation-set and test-set. The evaluation metrics are those presented in Section 6 and we also report the number of parameters and the processing speed (frames per second) for each combination.

NYUV2-test									
**Encoder**	**Decoder**	**#param.**	**Abs_Rel**	**Sq_Rel**	RMSE	$\delta_{1}$	$\delta_{2}$	$\delta_{3}$	**fps**
ViT-b16	US1	94.47M	0.0263	0.03906	0.0340	0.9986	0.9998	0.9999	10.97
ViT-s16	US1	51.94M	0.0401	0.09450	0.0511	0.9940	0.9988	0.9997	15.87
ViT-t16	US2	35.42M	0.0398	0.0871	0.0508	0.9930	0.9985	0.9997	18.18
ViT-t16	Deconv	34.94M	0.0308	0.0715	0.0447	0.9918	0.9984	0.9996	19.61
ViT-t16	DS	34.26M	0.0444	0.1151	0.0583	0.9893	0.9979	0.9997	20.83
**Cityscapes-val**									
**Encoder**	**Decoder**	**#param.**	**Abs_Rel**	**Sq_Rel**	RMSE	$\delta_{1}$	$\delta_{2}$	$\delta_{3}$	**fps**
ViT-b16	US1	94.48M	0.0932	1.2722	1.2242	0.9122	0.9543	0.9682	8.53
ViT-s16	US2	49.61M	0.1811	1.9127	1.1807	0.7912	0.9120	0.9517	10.87
ViT-s16	Deconv	49.13M	0.0839	1.2222	0.9114	0.9196	0.9561	0.9679	11.11
ViT-t16	US2	35.44M	0.0935	1.2682	1.1820	0.9158	0.9560	0.9681	11.76
ViT-t16	Deconv	34.95M	0.0755	1.1487	0.9378	0.9278	0.9589	0.9694	12.05
ViT-t16	DS	34.27M	0.1594	1.0894	0.8511	0.7960	0.9243	0.9685	12.50
**Cityscapes-test**									
**Encoder**	**Decoder**	**#param.**	**REL**	**Sq Rel**	RMSE	$\delta_{1}$	$\delta_{2}$	$\delta_{3}$	**fps**
ViT-b16	US1	94.48M	0.2074	2.2867	1.2081	0.7287	0.8698	0.9305	8.53
ViT-s16	US2	49.61M	0.1811	1.9127	1.2400	0.7912	0.9119	0.9517	10.87
ViT-s16	Deconv	49.13M	0.2302	2.6908	0.9466	0.6970	0.8428	0.9118	11.11
ViT-t16	US2	35.44M	0.2154	2.3763	1.2551	0.7376	0.8774	0.9338	11.76
ViT-t16	Deconv	34.95M	0.2156	2.4953	1.0759	0.7181	0.8601	0.9232	12.05
ViT-t16	DS	34.27M	0.1657	1.2547	0.8721	0.7900	0.9205	0.9646	12.50

**Table 2:** Comparison between the proposed method (specifically ViT-b16+US1 and ViT-t16+DS) and the SOTA methods on NYU-depthv2 depth estimation validation-set with respect to the Abs_Rel, $RMSE_{log}$, and the delta accuracies ($\delta_{1}$, $\delta_{2}$, and $\delta_{3}$).

Method	Abs_Rel	${\mathrm{RMSE}}_{\log}$	$\delta_{1}$	$\delta_{2}$	$\delta_{3}$
Eigen et al. [1]	0.158	0.641	0.769	0.950	0.988
Laina et al. [30]	0.127	0.573	0.811	0.953	0.988
Xu et al. [5]	0.121	0.586	0.811	0.954	0.987
Liu et al. [3]	0.127	0.506	0.836	0.966	0.991
Fu et al. [7]	0.115	0.509	0.828	0.965	0.992
SharpNet [31]	0.139	0.502	0.836	0.966	0.993
Chen et al. [1]	0.111	0.514	0.878	0.977	0.994
BTS [13]	0.110	0.392	0.885	0.978	0.994
DPT-Hybrid [25]	0.110	0.357	0.904	0.988	0.998
Zhang et al. [32]	0.144	0.501	0.815	0.962	0.992
SDC-Depth [29]	0.128	0.497	0.845	0.966	0.990
AdaBins [22]	0.103	0.364	0.903	0.984	0.997
PhaseCam3D [12]	0.093	0.382	0.932	0.989	0.997
**ViT-b16+US1**	0.026	0.034	0.998	0.999	0.999
**ViT-t16+DS**	0.044	0.058	0.9893	0.998	0.999

**Table 3:** Comparison between the proposed method (specifically ViT-s16+Deconv and ViT-t16+DS) and the SOTA methods on the CITYSCAPES depth estimation test set with respect to the Abs_Rel, $RMSE_{log}$, and the delta accuracies ($\delta_{1}$, $\delta_{2}$, and $\delta_{3}$).

Method	Abs_Rel	${\mathrm{RMSE}}_{\log}$	$\delta_{1}$	$\delta_{2}$	$\delta_{3}$
Laina et al. [29,30]	0.112	4.771	0.850	0.938	0.966
Xu et al. [5,29]	0.246	7.117	0.786	0.905	0.945
Zhang et al. [29,32]	0.234	7.104	0.776	0.903	0.949
SDC-Depth [29]	0.227	6.917	0.801	0.913	0.950
**ViT-t16+Deconv**	0.0755	1.0759	0.9278	0.9589	0.9694
**ViT-t16+DS**	0.1694	0.8721	0.7900	0.9205	0.9646

**Figure A1:**
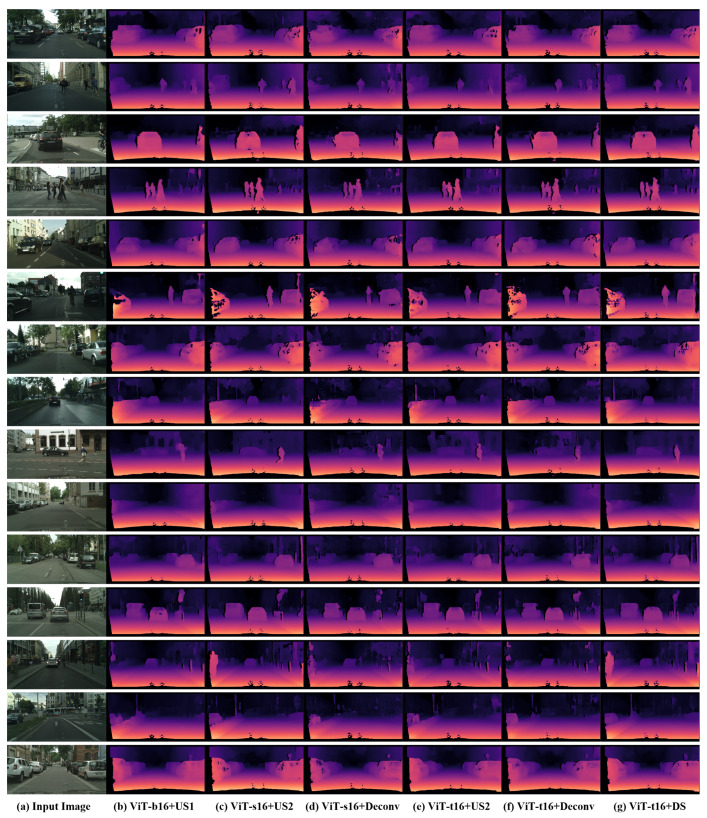
Sample results obtained from the proposed architectures on Cityscape validation-set: (**a**) the input image, (**b**–**g**) are the depth estimation results obtained from the architecture mentioned below the column, (**b**) ViT-b16 encoder and US1 decoder, (**c**) ViT-s16 encoder and US2 decoder, (**d**) ViT-s16 encoder and the Deconv decoder, (**e**) ViT-t16 encoder and US2 decoder, (**f**) ViT-t16 encoder and the Deconv decoder, and (**g**) ViT-t16 encoder and (depth-to-space) DS decoder.

## Data Availability

The datasets used in this paper are public datasets. We also provide the test and the evaluation codes of the proposed method at: https://github.com/HatemHosam/RT-ViT-Real-time-vision-transformer, accessed on 30 December 2021.

## Appendix A

Extra qualitative depth estimation results on Cityscapes dataset are obtained, those results are obtained on Cityscapes testset that are shown in Figure A1.
